# Comment on “Expanding horizons in burn care: a new paradigm for General Surgeons in Brazil”

**DOI:** 10.1590/0100-6991e-20243825-en

**Published:** 2024-12-02

**Authors:** VOLNEY PITOMBO

**Affiliations:** 1 - Sociedade Brasileira de Cirurgia Plástica - São Paulo - SP - Brasil

The article entitled “Broadening horizons in burn care: a new paradigm for General Surgeons in Brazil”^1^, sent by members of the Board of Directors of the Brazilian Society of Burns (SBQ), brings to light a relevant discussion about the care of burn patients in our country. Although the proposal to train general surgeons to treat burns may initially seem like a solution to expand access to treatment, it is crucial that the proper management of burn patients be conducted, in all phases, by plastic surgeons, specialists trained to manage the complexity of these injuries.

Burn patient care goes far beyond initial wound management. The expertise of plastic surgeons ranges from emergency treatment, through advanced grafting, flap, and tissue reconstruction techniques, to functional and aesthetic rehabilitation. These are essential aspects for the patient’s full recovery and are often neglected when the treatment is not conducted by professionals with specific training.

The training of the plastic surgeon includes extensive preparation in tissue reconstruction techniques, essential in the management of severe burns, and in the ability to prevent and treat the complications that often arise during recovery, such as scar contractures and deformities. In addition, the evaluation of the burn patient must consider both functional and aesthetic aspects, which have a profound impact on the individual’s quality of life. Plastic surgeons are trained to offer a comprehensive care that aims to minimize sequelae and maximize the patient’s physical and psychological recovery.

Although it is understandable that in some regions of Brazil, particularly those farther from large centers, there is difficulty in accessing specialized units, the treatment of burns should not be simplified to the point of compromising the standard of care. The focus should be on creating a regional referral network, where cases can be quickly transferred to specialized centers with multidisciplinary teams, led by plastic surgeons. This way, the burn patient will receive the appropriate treatment from the beginning, without compromising their recovery due to lack of expertise.

In addition, the complexity of burn treatment is not limited to the initial moment of care. Often, the patient’s recovery requires multiple surgeries over months or years, and follow-up by professionals who have deep knowledge about the behavior of scars and the functional impact of these injuries is crucial.

Allowing general surgeons to take over the treatment of burns can generate a false sense of solution to the problem of lack of access, when in fact the patient would be deprived of specialized care, often indispensable to avoid severe complications. The development of partnerships between plastic surgeons and general hospitals, with specific training on screening and stabilization of burns before referral, would be a more effective solution to ensure initial care without losing the standard of quality of treatment.

In conclusion, the care of burn patients should remain under the responsibility of plastic surgeons, professionals who have the necessary training and experience to deal with the complexity of this type of injury. The decentralization of care, although possibly beneficial in some cases, should not compromise quality, and it is essential that burn patients have access to the most advanced surgical treatment and rehabilitation. Maintaining the role of plastic surgeons on the front line of the management of these lesions is, therefore, essential to ensure the best possible prognosis for these patients.
